# Navigating the complexity of AI adoption in psychotherapy by identifying key facilitators and barriers

**DOI:** 10.1038/s44184-026-00199-1

**Published:** 2026-03-07

**Authors:** Julia Cecil, Insa Schaffernak, Danae Evangelou, Eva Lermer, Susanne Gaube, Anne-Kathrin Kleine

**Affiliations:** 1 Department of Psychology, LMU Center for Leadership and People Management, LMU Munich, Munich, Germany; 2 Department of Business Psychology, Technical University of Applied Sciences Augsburg, Augsburg, Germany; 3 UCL Global Business School for Health, University College London, London, UK

**Keywords:** Health care, Social sciences, Psychology

## Abstract

Artificial intelligence (AI) technologies in mental healthcare offer promising opportunities to reduce therapists’ burden and enhance healthcare delivery, yet adoption remains challenging. This study identified key facilitators and barriers to AI adoption in mental healthcare, precisely psychotherapy, by conducting six online focus groups with patients and therapists, using a semi-structured guide based on the NASSS (Nonadoption, Abandonment, Scale-up, Spread, and Sustainability) framework. Data from *N* = 32 participants were analyzed using a combined deductive and inductive thematic analysis. Across the seven NASSS domains, 36 categories emerged. Sixteen categories were identified as factors facilitating adoption, including useful technology elements, the customization to user needs, and cost coverage. Eleven categories were perceived as barriers to adoption, encompassing the lack of human contact, resource constraints, and AI dependency. Further nine, such as therapeutic approach and institutional differences, acted as both facilitators and barriers depending on the context. Our findings highlight the complexity of AI adoption in mental healthcare and emphasize the importance of addressing barriers early in the development of AI technologies.

## Introduction

The treatment gap in mental healthcare, resulting from a discrepancy between the need for treatment and its availability, remains a significant global challenge^1,2^. One in eight people is likely to develop a mental disorder during their lifetime^3^. However, with a global median of only 13 mental health professionals per 100,000 people, each would theoretically be responsible for over 7000 individuals, including approximately 960 requiring care^3^. This shortage is reflected in long waiting times, for example, in Germany, patients wait an average of 14.5 weeks for an initial appointment^4^, with other countries showing similar patterns^5^. This unrealistic demand for and shortage of mental health professionals, often coupled with inadequate coordination among psychotherapy providers, hinders optimal care^2,6^. Several additional factors contribute to inadequate treatment, including access barriers (e.g., travel costs), fear of stigmatization, and the misinterpretation of mental health symptoms as normal responses to social and economic challenges, rather than as treatable conditions^2^.

The advent of digital health technologies enabled by artificial intelligence (AI) presents new opportunities to help bridge the treatment gap and transform mental healthcare. AI-enabled technologies include tools for screening, diagnosing, predicting, and treating mental health conditions, as well as solutions designed to enhance therapists’ performance and streamline administrative tasks^7–10^. While technologies for screening, diagnosing, feedback, and practice management primarily focus on decision support and efficiency improvements, AI-enabled treatment applications actively engage both patients and therapists in the psychotherapeutic process. This paper focuses on the latter category of applications within the psychotherapeutic treatment process, emphasizing their dynamic interaction with both user groups - a feature that distinguishes them from other application areas. These technologies leverage advanced algorithms to customize treatment^11^. For instance, they can support therapists’ work by providing psychoeducation or therapeutic interventions specifically tailored to individual patient’s needs^12^. Oftentimes, these AI-enabled treatment tools focus on depression and anxiety^8,11^. For instance, natural language processing (NLP) models can assist in selecting the most effective therapeutic approach for treating depression, such as cognitive behavioral therapy (CBT) or drug treatment, tailored to the individual patient^13^. Overall, AI-enabled technologies have the potential to enhance the therapists’ work experience by reducing assessment times^14^, while providing improved and more timely support for patients^14,15^. Such improvements for patients and the ability to predict therapeutic responses can, for example, be facilitated through personalized approaches to treatment, commonly referred to as precision medicine. This paradigm tailors interventions to the unique biological, environmental, and lifestyle characteristics of each patient, thereby enabling more accurate and individualized therapeutic decision-making^16^. AI is particularly valuable in this context, as it can efficiently process complex health data to support, for instance, precise risk assessment and the selection of optimal therapies^16,17^.

Despite these possibilities, both patients and therapists demonstrate reservations about these technologies. Mental health professionals show a low intention to use AI-enabled technologies, especially patient-centered tools, including those used for treatment^10,18^. Among patients, research has demonstrated that dropout rates for AI-enabled psychoeducation applications, for example, range from 5 to 34%^19^. This reluctance might highlight a critical issue: much of the existing research on technology adoption in psychotherapy focuses on the design and functionality of AI applications rather than addressing user needs^20–25^. Understanding user needs and perceptions towards AI technologies is crucial to identify, address, and ideally prevent barriers that could hinder appropriate technology adoption.

**Fig. 1:**
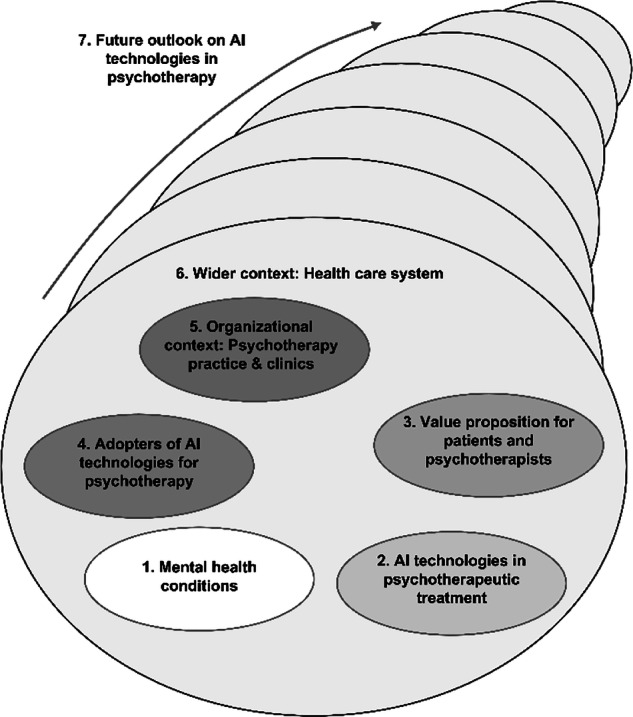
The NASSS framework specified for AI in psychotherapeutic treatment, adapted from Greenhalgh et al.^26^.

Recognizing that many healthcare innovations fail due to a variety of factors, the Nonadoption, Abandonment, and Challenges to the Scale-up, Spread, and Sustainability (NASSS) framework was developed by Greenhalgh et al.^26^. It provides a structural approach to analyze multiple dimensions and complex dynamics shaping the use of AI in healthcare by capturing barriers and facilitators to technology adoption in seven domains (see Fig. 1). In mental healthcare, the NASSS framework has been used to guide adoption research on video therapy consultations^27^, and internet-delivered CBT, and CBT in virtual reality^28^.

The first domain, *condition(s) or illness*, focuses on the nature and complexity of health conditions targeted by the technology in question^26^. It further encompasses clinical and sociocultural aspects, such as comorbidities^26^. Research on the adoption of AI technologies designed for mental disorders, such as depression or eating disorders, has mostly focused on already developed specific applications^20,25^. However, little research takes a bottom-up approach by first exploring which disorders users consider suitable or not for AI treatment tools.

The second domain, *technology or technologies*, refers to the technology’s characteristics, including their features, functionality, and usability^26^. An established requirement for AI adoption in mental healthcare is the technology’s foundation in robust scientific evidence and its capacity for personalization to meet patient’s specific needs^20,29^. Interestingly, specific features vary in relevance. For instance, tracking and feedback are generally viewed positively, while features like goal setting and relaxation audios are less preferred and may pose barriers to adoption^30^.

In the next domain, *value proposition*, concerns and benefits, such as the perceived desirability, efficiency, and cost-effectiveness of the technology, are examined^26^. From an economic perspective, some applications, such as AI-enabled computer assisted therapy, have been shown to be cost-efficient^31^. However, the use of digital technologies for the diagnosis and treatment of depression, does not seem to lead to healthcare cost savings^32^.

The fourth domain, *adopter system*, includes users’ perceptions of adoption and workload considerations, addressing factors such as training needs and individual-level barriers to adoption. On the therapist side, these issues include insufficient training, both before and during the adoption of AI technologies^29,33,34^. Additionally, there are concerns about the dehumanization of care and perceived power imbalances between therapists and patients due to shifting dynamics in the triadic relationship of therapists, patients, and technology^29^. From the patient’s perspective, key barriers also include a perceived loss of human connection and lack of professional oversight^35,36^.

Next, the domain *organization(s)* address the readiness and capacity of an organization for a specific technology^24^. Although limited research addresses barriers at this level in mental healthcare, financial challenges hindering AI adoption were identified in other healthcare contexts^37,38^. Specifically, a lack of funding, costs associated with expert input for labeling and curating AI training data, and technology maintenance create significant barriers to adoption at the organizational level^37,38^.

The sixth domain, *wider context*, considers the structure, dynamics, and capacity of the healthcare system in which the innovation is introduced^26^. The domain examines how the new technology interacts with existing practices, policies, and resources. Prior research on AI in psychotherapy identified obstacles such as cost coverage^35^ and regulatory issues, specifically the absence of structured implementation frameworks^29,33,34^.

The final domain, e*mbedding and adaptation over time*, addresses the challenges and strategies associated with expanding the reach and impact of the innovation beyond initial pilot or research settings^24^. This domain has received limited attention in research^37,39^; however, some factors, such as ongoing technology maintenance seem to play a role^38,40^.

Despite a growing body of research on AI applications in mental healthcare, a gap remains in understanding the adoption barriers from a bottom-up perspective, particularly given the limited findings in this specific field of healthcare. This study, therefore, investigates the needs and barriers from both patient and therapist perspectives through the NASSS framework, addressing areas typically explored through a top-down lens, such as organizational and system-level structures. In doing so, we aim to generate insights that can inform best practices for stakeholders, including developers, helping to proactively address user reservations prior to the development of AI technologies.

## Methods

### Study design

This qualitative study used focus groups to explore the needs of both patients and therapists for AI-enabled technologies in psychotherapy. Focus groups provide an opportunity for an exploratively in-depth analysis of an area with limited prior research and enable nuanced, contextual understanding of participants’ perspectives and underlying reasoning^41^. This pre-registered study (https://osf.io/9rt52) received ethical approval from the Gemeinsame Ethikkommission der Hochschulen Bayerns (GEHBa; English: Joint Ethics Committee of Bavarian Universities), identifier: 2023-11-V-144-R. It adheres to the 32-item Consolidated Criteria for Reporting Qualitative Research guidelines^42^, see supplementary material on OSF.

### Recruitment and participants

The study included two participant groups: therapists and patients. Therapists were recruited via emails distributed among psychotherapy training institutions and patients were recruited via flyers in and distribution channels of two German universities and two German patient organizations. Therapists were required to be licensed or undergoing psychotherapy training. Patients were required to be enrolled in psychotherapy and to be diagnosed with either depression or anxiety disorder. These conditions were chosen due to their high global prevalence and burden^43^.

A total of 32 participants were recruited, comprising 19 therapists and 13 patients. Participant characteristics can be found in Table 1. Each focus group consisted of four to six participants, with therapists and patients placed in separate groups. Using homogenous groups enhances the external validity of the research as it allows for in-depth discussions between participants, providing each group with space tailored to their specific experiences and perspectives^44^. Additionally, this approach helps mitigate any potential power imbalances between therapists and patients, creating a more open and balanced discussion environment^44^.

**Table 1:** Sample demographics

	Patients (*n* = 13)	Therapists (*n* = 19)
	*N* (%) or *M* (*SD*)	*N* (%) or *M* (*SD*)
Age	30.23 (10.05)	33.32 (10.55)
Gender		
Female	10 (76.9%)	15 (79.0%)
Male	2 (15.4%)	4 (21.1%)
Non-binary/third gender	1 (7.4%)	
Mental disorder		
Depression	10 (55.7%)	
Anxiety	5 (27.8%)	
Other	3 (16.7%)	
Profession		
Therapist in training		17 (89.5%)
Therapist		2 (10.5%)
Therapeutic approach		
(Cognitive) behavioral		12 (57.14)
Psychodynamic		6 (28.57)
Psychoanalytic		2 (9.52)
Systemic		1 (4.76)
Workplace		
Practice		3 (10.34)
Private practice		1 (3.45)
Specialist hospital for psychiatry, psychotherapy, psychosomatic medicine or neurology		7 (24.18)
Rehabilitation clinic		4 (13.79)
(University) outpatient clinic		10 (34.48)
Community mental health center/ counseling center		1 (3.45)
Other		3 (10.34)
Professional experience (in years)		3.89 (4.78)

*Note*. Combined age for therapists and patients: *M*_all_ = 32.06, *SD*_all_ = 10.3

### Data collection

Data was collected between March and May 2024. The online focus groups were conducted in German and via Zoom (Zoom Video Communications, Inc.), and lasted 58.36 min on average (SD = 2.05, min = 56.23, max = 61.49). Participants signed a data protection and consent form prior to their participation. Additionally, they received a general description of AI and AI-enabled technologies in psychotherapy to facilitate their familiarity with the subject in advance as follows: “Artificial Intelligence (AI) refers to computer programs and systems that can perform tasks which normally require human intelligence by analyzing data, recognizing patterns, and learning autonomously. In psychotherapeutic treatment, AI-powered technologies can take over certain elements of therapy, thereby reducing the workload of practitioners, among other benefits. These elements include, for example, psychoeducation and self-help exercises provided through apps or chatbots, which can be tailored to patients’ needs using algorithms. This allows patients to strengthen their resources outside the therapy room while also documenting symptoms and behaviors. The algorithmic analysis of patient data further enables personalized treatment recommendations and adjustments to the existing therapy plan”. The description and broader lens were chosen to offer a general overview of the various tasks AI can perform in psychotherapy, capturing considerations that cut across different AI applications in psychotherapeutic treatment rather than concentrating solely on a specific tool. The description alongside the original German description can be found in the supplementary material on OSF. The description of AI in psychotherapy was sent to participants together with the informed consent form, which had to be signed prior to participation, ensuring that all participants received the information in advance. At the beginning of each focus group, participants were informed about the purpose and procedure of the discussion and that their participation was voluntary and could be withdrawn at any time. The focus groups proceeded with a brief recap of AI-enabled technologies in mental healthcare to reinforce participants’ understanding by describing the overall function of AI and AI in psychotherapeutic treatment as in the priorly sent description but additionally adding the following part: “Overall, AI-enabled technologies offer a flexible, accessible, and effective way to support patients in improving their mental health. By combining interactive modules, self-help exercises, guided meditations, and other tools, they can empower patients to utilize their own resources and become active participants in their recovery process. It is important to emphasize that AI-enabled systems in psychotherapy take over specific tasks, such as personalizing content, analyzing data, and generating recommendations. However, they should always be overseen and used by qualified professionals, as they cannot fully replace the human aspect of therapy” (see online Supplementary Information). The focus groups were facilitated by two moderators. One moderator guided the discussions, while the other posted the discussion guide questions in the chat and addressed participants’ questions. For the questions, a semi-structured discussion guide was used. The discussion guide questions are based on the NASSS framework^26^ and related studies in this field (for instance, see ref. ^28^). Questions and prompts only differed in the wording to address each groups’ distinct characteristics, see online Supplementary Information.

### Data analysis

The sessions were recorded and transcribed verbatim using Trint (Trint Limited). Content analysis was conducted with the transcripts using MAXQDA (VERBI Software). Data analysis followed the deductive and inductive approach outlined by Fereday and Muir-Cochrane^45^. First, a code template was deductively developed based on the theoretical structure of the NASSS framework, which provided seven broad coding domains^45^. The template contains the names of the domains with a definition and how to apply them to the transcripts. Second, to ensure the reliability of the codes, two researchers (JC and DE) familiarized themselves with the transcripts, and third, the transcripts were coded independently according to the respective seven domains of the NASSS. Overall, complete sentences were used as the smallest coding unit and multiple codes were applied to the same sections if necessary. Subsequently, an inductive approach was employed to identify emerging themes from the data, which were organized within these predefined NASSS domains. In this fourth step, two researchers coded the transcripts independently again. It is worth noting that, during this step, the original NASSS subcategories were coded when explicitly mentioned in the focus groups. However, participants predominantly generated new categories that exceeded existing NASSS subcategories. As a result, we opted not to constrain new categories to the predefined NASSS subcategories, ensuring that the analysis remained true to the participants’ contributions. Fifth, as new themes emerged, they were grouped together and assigned concise labels. Lastly, after the independent coding, both researchers compared their categories and the names of the categories were refined and merged, resulting in a final codebook. Both researchers then independently coded all transcripts again as part of the reflexivity process. The intercoder agreement after this step, measured as a Cohen *κ* value of 0.73 for segments with at least 90% overlap, indicates a moderate to substantial level of agreement^46^. Any discrepancies in coding during this step were discussed with a third researcher (IS) and resolved through consensus.

## Results

**Fig. 2:**
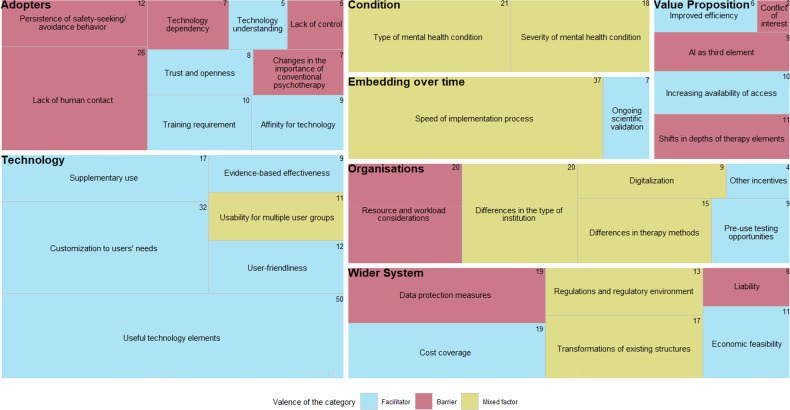
Tree map with categories regarding AI adoption in psychotherapy clustered along the NASSS domains. *Note*. Numbers on the upper right corner of each category indicate the total number of codes assigned to that category. Tile size reflects the frequency of codes.

The final codebook contained 561 codes, ranging from 83 to 102 codes per focus group. The seven domains of the NASSS framework, along with the emerging subcategories, their descriptions, and the proportion of references from therapists and patients, are summarized in Table 2. Each category of the NASSS was classified as a *facilitator or barrier* when at least two-thirds of the codes reflected a positive or negative influence on AI technology adoption, respectively. In cases when less than two-thirds of the codes reflected an overall positive or negative influence, the category was classified as *mixed*. We chose this rule as a descriptive aid to facilitate consistency in classification decisions and comparability across NASSS categories. The number of codes for each category and their valence (i.e., barrier, facilitator, or mixed) can be seen in Fig. 2.

**Table 2:** The NASSS domains, categories, and descriptions regarding the adoption of AI technologies in psychotherapy

NASSS domain	Category	Description	Therapist/Patient ratio
*Condition*		*The nature and complexity of mental health conditions targeted by AI technologies*.	
	Type of mental health condition	The specific mental health conditions that AI may target.	13/8
	Severity of mental health condition	The range of illness severity that AI may address.	7/3
*Technology*		*The characteristics of AI technologies*.	
	Useful technology elements	The specific elements that are considered useful for AI.	39/11
	Customization to users’ needs	The extent to which AI can be tailored to specific users’ needs.	17/15
	Supplementary use	The use of AI as an adjunct rather than a replacement for traditional therapy.	8/9
	Usability for multiple user groups	The extent to which AI is suited for use across different groups.	8/3
	User-friendliness	The ease and intuitiveness with which users can use AI.	8/4
	Evidence-based effectiveness	Evidence supporting AI’s effectiveness in improving mental health outcomes.	7/2
*Value proposition*		*The value of AI technologies for users*.	
	Increasing availability of access	The ability for users to access AI at their convenience.	6/5
	Improved efficiency	Potential improvements in efficiency offered by AI.	-
	Shifts in depths of therapy elements	The risk of AI oversimplifying therapy and reducing nuanced insights.	9/2
	AI as third element	The role of AI as an additional agent during sessions, affecting roles, responsibilities, and the structure of therapeutic interactions.	8/1
	(Commercial) conflict of interest	The influence of commercial interests in the adoption of AI.	1/1
*Adopters*		*User factors influencing the adoption of AI technologies*.	
	Affinity for technology	Users’ comfort level and enthusiasm in using AI.	8/1
	Training requirement	The need for training to use AI effectively.	9/1
	Lack of control	The potential loss of control over therapy processes due to AI.	4/1
	Trust and openness	The degree of trust in and openness for AI among users.	4/4
	Technology understanding	The knowledge or technical literacy required for effective use of AI.	4/1
	Lack of human contact	The absence of social and emotional connection between therapist and patient after the integration of AI.	13/13
	Changes in the importance of conventional psychotherapy	The potential of AI changing the perceived need for conventional therapy.	4/3
	Technology dependency	(Over-) Reliance on AI technologies.	3/4
	Persistence of safety-seeking/ avoidance behavior	The potential of AI to reinforce users’ avoidance of difficult interactions or situations.	11/1
*Organizations*		*The readiness and capacity of an organization for AI technologies*.	
	Differences in the type of institution	Underlying differences in the type of therapy-providing institutions influencing the use of AI.	17/3
	Differences in therapy methods	The compatibility of different therapeutic approaches with the potential use of AI.	13/2
	Digitalization	The extent of digital infrastructure present in institutions.	7/2
	Pre-use testing opportunities	The chance to try the AI at least once before use.	6/3
	Other incentives	Existing services or support structures necessary for adopting AI.	3/1
	Resource and workload considerations	The resources required for integrating AI.	-
*Wider system*		*The structure, dynamics, and capacity of the system in which AI technologies are introduced*.	
	Cost coverage	The extent to which the use of AI is covered by insurance or other funding sources.	12/7
	Economic feasibility	Economic considerations regarding the adoption of AI.	6/5
	Regulations and regulatory environment	Legal and policy frameworks guiding the use of AI.	11/2
	Data protection measures	Measures ensuring data protection for the use of AI.	13/6
	Liability	Determining accountability for AI outcomes.	3/3
	Transformations of existing structures	Shifts in the structures of the mental healthcare system.	9/8
*Embedding and adaptation over time*		*The adjustments in the adoption of AI technologies over time*.	
	Speed of implementation process	The time it takes to integrate and adopt AI.	15/22
	Ongoing scientific validation	The necessity of ongoing research to validate the efficacy of AI prior to its use.	6/1

### Condition

For the domain condition, two categories regarding the disorder characteristics emerged as mixed factors (labeled M).

For *Type* *of mental health condition (M)*, participants identified 15 mental health conditions that could potentially be addressed by AI technologies, with anxiety disorders and depression being the most frequently mentioned. Participants generally agreed on the potential usefulness of AI technologies for the following conditions: panic disorder, sleep disorder, hyperkinetic disorders, conduct disorders, substance use disorders, gender dysphoria, and borderline personality disorder. For instance, one patient explained:

*“If there’s […] a button: ‘I’m having a panic attack. Help me’, and it tells me to do different exercises or whatever, I would take it.” (PA01)*.

Opinions were more divided for anxiety disorders, depression, eating disorders, ADHD/ADD, and suicide. For instance, therapists highlighted both potential benefits and risks of AI-based data tracking for eating disorders. Tracking physical activity and identifying deviations from usual activity patterns could provide valuable insights, as patients might not be fully aware of behaviors like excessive walking, which could contribute to difficulties in weight gain. However, such tracking could become a new obsession for patients, leading to an unhealthy focus on monitoring behaviors.

Participants were skeptical about using AI technologies for conditions such as social phobia, psychosis, and paranoia. For instance, one therapist remarked that patients with paranoia or psychosis might be even more reluctant to adopt AI technologies, as these tools could amplify their existing suspicion and mistrust of their surroundings, leading to heightened discomfort and anxiety.

For *Severity of mental health conditions (M)*, participants emphasized the need to distinguish between mild, moderate, and severe mental health conditions to ensure appropriate adoption of AI. AI was unanimously deemed useful for milder conditions, but participants were hesitant about its application in more severe cases. They suggested a cautious, stepwise approach, starting with mild conditions and gradually expanding to more severe conditions.

### Technology

In the technology domain, five categories were perceived as facilitators (labeled F) and one as mixed, portraying the AI functionality.

For the category of *Useful technology elements (F)*, participants had various ideas for beneficial technology elements that would facilitate the adoption of AI technologies. Table 3 presents an overview of the designated technology features.

**Table 3:** Useful technology elements retrieved from the focus group discussions

	Feature
*Information purposes*	
	Psychoeducation
	General information
*Emotional and behavioral management*	
	Emotion regulation
	Mood tracking
	Mood detection
	Skills^a^
	Relaxation exercises
*Logging and monitoring/ tracking*	
	Triggers
	Different types of diaries
	Activity tracking
	Recording of critical events
	Identifying patterns^a^
*Therapist support and process analysis*	
	Data analysis for therapists^a^
	Analysis of the therapy process
	Monitoring the development of the therapeutic relationship
	Decision support for treatment options
	Decision support for the therapeutic approach
*Self-management and organization*	
	Homework assignments
	Reminders
	Creating a daily structure
	(Self-help) Exercises
	Emergency plan
	Reinforcement plan/ system
*Safety and risk management*	
	Detection of suicidality
*Technical and interactive features*	
	Avatar/ representation of the therapist
	Automated insurance form completion
	Voicemail

^a^not specified further

The most prominent features included psychoeducational content, mood tracking, different types of diaries, and self-help exercises like imaginary journeys. For instance, the value of mood tracking beyond fixed points was highlighted, especially when daily routines are disrupted, to provide deeper insights into a patient’s emotional state.

Another critical factor for the adoption of AI technologies in mental healthcare is *Customization to users’ individual needs (F)*. Personalization might help alleviate symptoms, while its absence might even worsen them:

*“Every panic attack manifests itself differently, and what helps one person may not help another at all. You tell one person to concentrate on your breathing, and then they start to hyperventilate. You tell the other person to concentrate on your breathing and they calm down. […]” (PA03)*.

Further, for *Supplementary use (F)*, participants from both groups stressed using AI only as a supplement technology, not as a replacement for traditional therapy or a standalone solution, since they view human therapists as irreplaceable in the therapeutic process.

Regarding *User-friendliness (F), p*articipants stressed that the technology should be easy to use, especially to reduce cognitive and emotional strain for individuals who may already feel overwhelmed.

*“I think it should be very intuitive and not so complex, because especially if you have depression or something like that, you just don’t have the strength to get into it.” (PA08)*.

Finally, participants emphasized the need for *Evidence supporting the effectiveness* (F) of AI technologies in improving outcomes.

*“There should be studies that prove that AI-supported interventions have an effect, which is why it makes sense to use it, but the effect is not as great as psychotherapy from a real person.” (TH14)*.

Another AI adoption factor was its *Usability for multiple user groups (M)*. Three different sociodemographic areas were mentioned in which adoption barriers should be addressed: age, socioeconomic status, and culture and language:

First, older generations should be enabled to use emerging technologies.

*“It is important to have a different perspective for children and young people than for adults or geriatric patients. I can possibly see difficulties there […], due to their probably rather limited use of digital devices. In other words, I ask myself, are these older patients then excluded? That should, of course, be avoided.” (TH18)*.

Second, usage should be possible regardless of personal financial background, as participants emphasize potential inequalities for those with a lower socio-economic status.

And third, adaptations to different cultural and language backgrounds should be considered.

*“[…] I believe that there will also be a large section of the population that you do not deal with because it will then be very complicated to translate into simple language applications or actually offer something in their native language.” (TH11)*.

### Value proposition

In the domain value proposition, five categories emerged as either facilitators or barriers (labeled B) to AI adoption in mental healthcare, highlighting the impact of AI.

A key benefit of AI technologies is its *Increasing availability of access (F)*, both in terms of location and timing. Participants highlighted the advantage of using AI flexibly in different places and its critical role in timely supporting patients during moments of crisis by providing immediate access to therapy resources.

*“If I imagine: ‘Okay, I can fall back on previous therapy content, I’m in a crisis at the moment and can simply call it up’, I don’t necessarily need the therapist for that, that in turn can also have positive effects in terms of autonomy.” (TH18)*.

At the same time, one patient noted that increased accessibility through AI may not always be beneficial, as it cannot replace the structure and emotional containment provided by fixed, in-person therapy sessions.

Regarding *Improved efficiency (F)*, AI could enhance efficiency by automating certain tasks, such as data analysis and administrative processes, enabling therapists to focus on patient care and reducing the strain caused by high patient volumes and long waitlists.

For *Shifts in depths of therapy elements (B)*, concerns were expressed that AI technologies might oversimplify complex therapeutic dynamics and reduce the value of nuanced observation. AI might overlook interpersonal dynamics and subtle behavioral cues, which are essential for forming accurate therapeutic judgments.

*“[…] I wonder if […] [AI is] too simplistic because we [humans] are actually much more complex than that, more than could be broken down into an algorithm that works when certain words are written, like in chatbots or similar things. I mean, the risk that we overlook something or miss something […] is very high.” (TH02)*.

Further, for *AI as a third element (B)*, the potential drawback of AI as an additional agent in therapy was discussed, with concerns that it could disrupt the therapeutic relationship by causing confusion or conflicting interpretations, which can complicate the process and detract from the patient-therapist connection:

*“There is then a third element that may influence the therapeutic relationship. [….] Then we have triangulation, […], which I think is complex. In principle, it’s a bit like two therapists working on the same patient.” (TH01)*.

Another potential disadvantage might arise from *(Commercial) conflicts of interest (B)* in adopting AI technologies. Concerns were expressed about profit-driven motives and exploitation of sensitive data for commercial gain, as technologies are rarely developed by non-profit companies.

### Adopters

At the adopter level, nine distinct categories emerged as facilitators or barriers evolving around user characteristics.

Participants highlighted the importance of personal *Affinity for technology (F) for* its adoption, noting that individual user preferences affect how much they want to get involved in technology use and deal with its additional demands.

*Trust in and openness (F)* towards AI is crucial, especially in intimate or emotionally charged contexts, given the sensitive nature of the topics addressed in psychotherapy and the data shared with these technologies.

Regarding the *Training requirement (F)*, since integrating new technologies can be time-consuming, therapists and patients suggested that training, through videos or practical workshops, could be an essential support mechanism. This was considered especially important in smaller practices or clinics where staff need to be trained quickly to avoid staff shortfall.

For *Technology understanding (F)*, closely related to the need for training is the level of users’ knowledge or understanding to use AI technologies effectively. Therapists expressed the need to understand the technology to recommend it confidently to patients or to answer patients’ questions. While patients found it essential that the therapists understand the technology, they did not emphasize the importance of their own understanding.

*“[…] Therapists […] [should] also [be] familiar with it. So that they can also give me recommendations or perhaps explain how to use it. And that they’re not just at the level of saying: ‘Well, there’s this app, you can use it, it’s good, but I don’t really know much else about it’.” (PA06)*.

Regarding *Lack of control (B)*, therapists were concerned about the potential loss of control in the therapeutic process, such as their influence on the patient. Integrating AI into psychotherapy raised questions about maintaining control and ensuring it aligns with the therapist’s approach and methods.

Another emerging concern for both user groups was the *Lack of human contact (B)*, especially the absence of social and emotional connection. The loss of human connection, which is generally perceived as a main mechanism in psychotherapy, may hinder patients’ ability to open up and engage in meaningful and healing therapeutic work.

*“So, I wouldn’t feel seen or acknowledged at all because I would just know: ‘Okay, it’s just an algorithm.’ Artificial intelligence or not. What I would miss is the living counterpart, emphasis on living.” (PA03)*.

Further, for *Changes in the importance of conventional psychotherapy (B)*, concerns were expressed that integrating AI could diminish the perceived value of and demand for in-person psychotherapy, leading to fewer therapy slots and steering patients away from traditional, personalized care.

*“I can only put it plainly: there’s more of a concern that they’ll think, ‘Oh, great. A cheap, easy, and quickly available alternative - then we can cut costs elsewhere’.” (PA03)*.

Regarding *Technology dependency (B)*, both therapists and patients were concerned about the potential for technology over-reliance. With its constant availability, there are fears that it could lead to an unhealthy dependence on devices rather than fostering real-world coping mechanisms.

*“What I mentioned earlier as a pro argument could also be seen as a con argument here: the shift from independence from the therapist to dependence on a device.” (TH18)*.

Finally, for *Persistence of safety-seeking and avoidance behavior (B)*, the use of such technologies might reinforce avoidance or safety behaviors in patients. The ease of using AI, especially for patients with social anxiety or phobias, could encourage avoidance of real-world challenges, hindering therapeutic improvements. While AI may offer immediate relief by eliminating the need for human interaction or enhancing perceived control through safety behaviors such as using conversational agents, it risks distracting from deeper emotional work.

*“But it can perhaps also lead to patients starting to avoid contact or the relationship difficulties that only become apparent in therapy through shared experience, so that they don’t arise in the first place.” (TH02)*.

### Organizations

At the organizational level, six categories emerged as either facilitator, barrier, or mixed factor, either enabling or hindering organizational readiness.

Regarding *Pre-use testing opportunities (F)*, opportunities to interact with and test AI technologies prior to their use are essential. Such opportunities can be created, for instance, through organizational support from the top down. This would allow individuals to test the technology in a neutral setting before using it with patients.

Regarding *Other incentives (F)*, both patients and therapists believed that additional incentives should be introduced to support the integration of AI technologies into psychotherapy, such as a dedicated hotline for addressing questions that arise during and after the implementation process.

Regarding *Resource and workload considerations (B)*, therapists expressed concerns that AI integration would initially create more effort than relief, with the required resources, particularly the time investment, frequently described as a barrier. Hence, they stressed the need for AI adoption to cause minimal additional workload, considering the already demanding nature of their profession.

*“For me, […] this familiarization period would be very, very important, because I think it’s very important for us, but above all, for the patients, that you don’t somehow spend a whole hour of therapy on it [the familiarization period], so to speak, to deal with it first.” (TH17)*.

Regarding *Differences in therapy methods (M)*, therapists usually specialize in a specific therapeutic approach, which may vary in compatibility with AI technologies. AI might be more easily integrated into structured approaches like CBT, where predefined methods and tasks are common. However, more dynamic and individual approaches, such as psychodynamic or systemic therapies, may face challenges since their change mechanisms rely on countertransference, childhood experiences, and deeper emotional engagement.

*“I believe that behavioral therapy has far fewer problems than psychodynamic psychology in the whole complex that is being discussed here. And I think psychoanalysis is probably completely excluded.” (TH06)*.

Although one therapist challenged this consensus by noting that AI could still offer opportunities for diagnostics in psychoanalysis, even if practical implementation remains uncertain.

The *Differences in the type of institution (M)* providing therapy also impact AI adoption, as therapists work in diverse settings from private practices to large clinics. Clinics could be more inclined to adopt AI technologies, as they tend to have the resources and infrastructure to integrate new technologies more easily. However, adoption decisions may also be influenced by financial constraints, administrative structures, and the overall attitude within the institution.

*“At least in my clinic I can imagine that the clinic management or the head physician could be interested in this, because they tend to have such a market-oriented awareness and say that innovations and things like that are good and can certainly be incorporated.” (TH11)*.

However, the downsides of AI adoption in clinics with top-down decision-making were also discussed:

*“So, if I work in a clinic or in an outpatient clinic that I don’t manage myself, so to speak, then I’m dependent on the decisions of the person in charge. Whereas if I have my own outpatient practice, then I’m more independent and can introduce it more easily.” (TH18)*.

Further, participants felt that *Digitalization (M)* in psychotherapy is not yet advanced enough for the introduction of AI tools. Concerns were raised about the slow digital transformation in healthcare, with many institutions still lacking basic infrastructure like Wi-Fi.

*“I believe that we haven’t yet reached the point in the development of digitalization where it really saves time, where it [technology adoption] becomes automatic and where people have internalized it in such a way that they can say: ‘Okay, it [technology adoption] makes my work easier.” (TH09)*.

### Wider system

At the wider system level regarding the system readiness, six categories emerged as either facilitator, barrier, or mixed factor.

The prevailing topic was *Cost coverage (F)*. Therapists expressed the need for reimbursement strategies, and both user groups advocated for standardized insurance practices to ensure equitable access for patients. Although the majority of participants viewed cost coverage in principle as a facilitating factor for AI adoption, some concerns were raised regarding differing cost structures across applications, for example the higher costs associated with AI-enabled virtual reality tools.

For *Economic feasibility (F)*, according to participants, the coverage of development and maintenance costs of AI systems must be ensured to facilitate their adoption. Further, it was noted that economic feasibility should not be pursued at the expense of quality and long-term value.

The issue of *Liability* (B) for AI outcomes emerged as another concern, highlighted by a hypothetical scenario.

*“Let’s say I have suicide prevention software, and it somehow works better, ten times better than a therapist […] [in detecting risks]. But still, sometimes people die by suicide. The question then is: Who is somehow liable? Who bears the risk?” (TH08)*.

Regarding *Data protection measures (B), p*articipants expressed significant concerns about data security, emphasizing risks related to data breaches, unclear data processing, and storage. For example, in relation to chatbots, concerns were raised about the use of personal data, particularly when user input is stated to be used to improve services.

Further, regarding the category *Regulations and regulatory environment (M)*, regulations to guide the use of AI in psychotherapy were deemed necessary. Participants emphasized the importance of clear legal frameworks, both nationally and internationally, through centralizing regulatory efforts to ensure data protection and quality standards and prevent misuse of sensitive information. However, some voiced concerns about overregulation hindering flexibility.

*“If this is always linked to any guidelines, then it may be that the whole thing becomes quite a cage as far as the application is concerned.” (TH09)*.

For *Transformations of existing structures (M), p*articipants from both groups emphasized the need for a thoughtful approach between using AI to complement existing therapies and questioning whether its use would improve care or just address supply shortages short-term.

*“But we could perhaps become even faster. We could become even more productive. Perhaps we could see even more patients. Treat even more. […] Whether that is necessarily a good thing remains to be seen.” (TH10)*.

### Embedding and adaptation over time

Regarding the embedding and potential adaptations over time, two categories were derived, reflecting the adoption trajectory.

Regarding *Ongoing scientific validation (F)*, participants highlighted the necessity of ongoing research to validate the efficacy of AI technologies. While initial studies exist, the consensus was that widespread implementation requires constant research, including pilot projects and rigorous testing to meet clinical standards.

Opinions were mixed regarding the *Speed of the AI implementation process (M)* into psychotherapy. Some participants acknowledged the unpredictability of technological advancements, suggesting that the adoption process could proceed rapidly. This is particularly true for simpler and more general AI technologies, such as those for text or image processing, which are projected to be implemented within the next three to five years. However, they anticipated that more specialized and niche applications, such as standalone AI-enabled technologies for VR applications in exposure therapy, take longer to implement, approximately ten years. Further, systemic challenges, including legislative and funding uncertainties, were highlighted as significant obstacles to AI implementation.

*“This will probably take decades until Germany might actually reach the point where they say: ‘OK, AI-supported psychotherapy, […] maybe’. But only if all the framework conditions are 100% perfectly balanced and clarified.” (PA05)*.

### NASSS extension—Time of use

As the aim of this research is to gain a comprehensive understanding of patients’ and therapists’ perspectives on the use of AI in psychotherapy, it is crucial to identify the stages and time points in the therapeutic process where technologies are perceived most beneficial. Therefore, we extend NASSS with “*time of use*” as a new domain.

Participants acknowledged AI’s *Use in diagnostics and first screening (M)*, thus its potential to conduct preliminary diagnostic evaluations by analyzing patient-completed questionnaires or assessments before therapist review. While therapists viewed it as useful to streamline workflows, concerns were raised about over-reliance, particularly for complex cases where personal history and symptom nuances may be overlooked.

Participants saw the potential of AI to offer therapeutic resources during *Waiting periods or appointment delays (M)*, enabling early engagement. However, some emphasized that, especially in the beginning, therapeutic relationships should include personal interaction, which AI cannot replicate.

Participants judged the *Use of AI technologies between therapy sessions (F)* positively, considering them as a tool to offer continuity and fill gaps in the process, such as providing tailored exercises.

Regarding *Use in aftercare (F)*, AI technologies could also serve as a valuable resource for post-therapy support, to maintain continuity, offer security, and ease the transition to self-management, particularly for patients feeling unprepared to navigate challenges independently. Participants highlighted its potential for relapse prevention and supporting the reduction of therapeutic contact.

## Discussion

With the rise of AI technologies and growing pressure on therapists and mental health systems, applying the NASSS framework to understand both patients’ and therapists’ perspectives offers valuable insights into the challenges and needs of AI adoption. Although patients and therapists represent distinct user groups with different backgrounds, their discussions revolved around largely overlapping topics, with clear differences in only two areas: only therapists highlighted the need to minimize AI-related workload due to limited time and resources, and only patients emphasized AI’s potential to improve efficiency. Notably, nearly half of all codes were allocated to the technology domain (such as its features and functionality) and the adopter domain (related to users’ role), suggesting these areas are of great interest. The discussion highlights the most frequently cited categories, grouped into four broad areas:

First, across the different NASSS domains, the findings indicate that *heterogeneity in psychotherapy*, including the specific *type of mental health condition* (in the *condition* domain), is an important factor influencing AI adoption. The positive attitudes toward AI technology for depression and anxiety disorders align with trends in research and technological development, which predominantly focus on these conditions (e.g.,^8,11,47^). The reluctance to consider AI technologies for more complex mental illnesses, such as psychotic disorders, aligns with their broader hesitation toward their use in *severe symptomatology*. In these cases, such as suicidality, hesitation was only alleviated when the AI was intended solely for supplementary support or early detection rather than for use during acute phases or as a standalone solution.

Heterogeneity at the organizational level, including different *therapeutic approaches*, also needs to be considered. Consistent with our findings, prior research suggests that therapists’ theoretical orientations influence their acceptance of new technologies, with psychoanalytic therapists showing lower adoption rates^48^. Similarly, we found that AI integration is more compatible with CBT due to its structured, manualized, and rule-based methodologies. Another key barrier in this context is the variation in *organizational and institutional environments* therapists work in. Therapists noted that in clinical settings, decisions regarding technology adoption are often outside their control and depend on other stakeholders, such as clinic management. Participants believed that while clinicians and management are more motivated to adopt technologies, administrative stakeholders tend to hinder adoption due to investment costs. In contrast, therapists in private practice have greater autonomy but face the challenge of managing adoption on their own.

Second, *user-specific human factors* also played a pivotal role in AI adoption, with the *lack of human contact* (*adopter* domain) emerging as an especially critical aspect. This supports previous research emphasizing the need to preserve the human element in patient-therapist interaction when adopting AI technologies, especially in the emotionally sensitive field of mental healthcare^35,49,50^. Such concerns stem from fears that AI could undermine authentic human interaction, potentially disrupting meaningful therapeutic relationships^33,51,52^. For instance, meaningful relationships are shaped by emotional shifts, like countertransference in psychodynamic psychotherapy. Therapists were hesitant that AI technologies could address these shifts appropriately, stressing the need for human responsiveness. The lack of this human element may hinder therapeutic progress, given the well-established importance of the therapeutic relationship in psychotherapy outcomes^53^. Despite the growing body of research on AI systems establishing a human bond and their promising advancements^54^, such as enhancing social presence in chatbot-based interactions to foster rapport^55^, our findings underscore the continued importance that patients and therapists place on human contact. Furthermore, AI’s role as a *supplementary tool*, a topic central in the *technology* domain, emerged as a key facilitator. Participants agreed that human therapists are irreplaceable in the therapeutic process, and that AI technologies, such as AI-enabled mental health apps suggesting interventions, should serve only as a supplement, aligning with existing literature^11,56–58^.

Moreover, *customization to users’ needs* is one strategy commonly mentioned in the *technology* domain, that can help address concerns about the lack of human connection. In our study, customization includes adapting to patients’ therapy experience and knowledge, which helps avoid repetition and tailors advice to prevent suggestions that may worsen the situation. This personalized approach might foster a sense of connection and relevance, thereby mitigating concerns that technology may hinder the human element of therapy. The importance of customization is reflected in our findings, as users viewed customization as a critical feature, in line with previous research^30^. While the literature often focuses on technology customization for patients, for instance, to strengthen the therapeutic relationship by personalizing the therapy style and avatar of chatbots^59^, less attention is given to therapists’ perspectives. Our therapists stressed that customization on their end involves aligning technologies also with their practice, ensuring it is tailored to specific tasks that support their work and maintain authenticity, without compromising their therapeutic framework. Ideally, tailoring the technology to each user’s needs enhances its *usability across different user groups*, a key facilitator of the *technology* domain highlighted in the literature, particularly regarding age^33,60^. Studies have found hesitations towards AI technologies in older adults^61^, while younger patients tend to show greater engagement with technologies^62^. However, our findings even extend this by identifying additional sociodemographic factors, such as the patient’s financial situation, cultural background, and language requirements.

Third, the *accessibility of AI technologies* is also a crucial consideration. Participants emphasized the benefit of continuous support and *flexible access* to therapy content in the *value proposition* domain, particularly across locations and times. Other research also underscores the positive perception of accessible AI technologies, such as those via smartphones, which can reach patients in high-risk situations^57^ and offer 24/7 support^63^. However, in our study, participants had a more nuanced view: they noted that constant accessibility could lead to *dependency* (*adopter* domain), making the absence of AI technology feel unsettling. This dependency could even exacerbate symptoms by fostering compulsive behaviors, such as feeling compelled to engage excessively with technology. As existing research primarily focuses on the harm of dependency on chatbots^64^, our findings extend this concern to AI applications in psychotherapy in general, emphasizing the broader risk. Additionally, both user groups stressed the risk that AI technologies, through individualized and non-confrontational interactions, may reinforce *safety-seeking or avoidance behavior* for certain *adopter* groups by reducing the exposure to therapeutically relevant challenges. In psychotherapy, such challenges – like deliberate ambiguous or paradoxical therapist responses – are often critical for emotional processing and behavioral change. This concern was especially noted in relation to anxiety disorders, particularly social anxiety. While initially, since it has been shown that individuals with social anxiety tend to disclose more information to conversational agents than to human counterparts^65,66^, this might seem beneficial by promoting communication and engagement, it might hinder therapeutic process by limiting therapeutic opportunities to engage in real social interactions^67^.

Fourth, different *structures surrounding AI adoption* also play a pivotal role. Having a robust *digital infrastructure* at the *organizational* level is the foundation of AI adoption, however, the current infrastructure in both clinical and private practice settings remains insufficient. While the importance of adequate digital infrastructure in healthcare is widely recognized^68^, in practice, Germany lags behind global leaders in the development of AI technologies for mental health disorders^8^ and other European countries in the digitalization of medical services^69^. This lack of favorable conditions compounds another *organizational* challenge identified by therapists: *resource and workload constraints*. Adopting new technologies requires time and resources, and both our findings and prior studies indicate that therapists are rather reluctant to engage with time-intensive digital implementations^34^. Dealing with poor digital infrastructure adds workload and complicates the AI adoption process. It also undermines other facilitators, such as *pre-use testing opportunities* (*organizational* level) and comprehensive *training* (on *adopters’* side), that have been linked to facilitating adoption^21,70,71^.

Further, financial and structural transformations at the *wider-system* level, such as *cost coverage*, could help overcome technology and adopter-level barriers, for instance, by ensuring accessibility for diverse user groups. Cost coverage by health insurance companies is a prerequisite for patients to adopt AI technologies^36^. Additionally, therapists noted the need for affordable technologies and reimbursement^72^. However, participants indicated that meeting cost coverage needs might necessitate a restructuring of current reimbursement mechanisms. Moreover, participants anticipated that system-level structures, such as patient referral pathways, would undergo a *transformation*, as AI could increase access to therapy, allowing for a larger number of patients to be treated. This, in turn, raised some concerns, especially regarding the treatment quality, with the risk that these changes might offer only a temporary increase in access without addressing the underlying systemic issues, making them a “quick fix” rather than a lasting solution, and psychotherapy becoming less patient-centered and rather driven by economic efficiency.

Finally, technology *embedding and adaptation over time* have been largely overlooked in existing literature^37,39^. The majority of our participants expressed uncertainty about the speed of the adoption process and refrained from making generalizations across AI technologies. Instead, they anticipate slower adoption for more specialized and niche applications, while simpler and more general AI technologies are believed to be implemented quicker.

Beyond adoption speed, however, temporal aspects also played a central role with regard to the timing of implementation. While the NASSS framework provides a comprehensive structure for understanding end-user needs and contextual factors across its domains, our findings indicate the potential theoretical refinement through the explicit incorporation of the temporal dimension of implementation. This extension foregrounds when, and at which stages of the therapeutic process AI technologies are perceived as most beneficial, highlighting the dynamic nature of implementation over time. By integrating this temporal perspective, the NASSS framework can better account for changing user needs, readiness, and contextual influences throughout adoption.

Our findings highlight some important implications for the development, design, and implementation of AI technologies and further research. First, addressing the specific technological features valued by patients and therapists can support adoption^73^. Particularly, features enhancing patient autonomy, such as psychoeducational content to aid patients in understanding their conditions, self-help exercises like meditation or guided imagery, and diaries, are deemed most useful. Notably, a common characteristic of these features is their utility when therapists are unavailable and during pre-therapy phases, including diagnostic and initial screening, or while awaiting treatment. Second, aligning the purpose of the AI technology with users’ preferred timing of use is critical to reduce hesitation. Participants in our study identified four key timepoints where they believed that AI could support and enhance the therapeutic process. At each of these stages – diagnostics and screening, before therapy, between therapy sessions, and in aftercare – AI’s use would need to be tailored to the specific needs and goals of that phase to be effective and well-received. Third, there was a substantial overlap in the topics discussed by patients and therapists. While addressing the distinct needs of each user group is essential for AI adoption, targeting shared barriers may simplify adoption and reduce complexity for both. Finally, our findings of mixed categories where the same aspect can be perceived as a barrier and facilitator in almost all NASSS domains highlight the complexity on every level of technology adoption. Understanding these dual-role factors is crucial for developing strategies that maximize their facilitative potential while minimizing drawbacks. For instance, the need for clear legal regulations for technology adoption was emphasized among participants and is also highlighted in the literature^7,29^. Regulations should clarify the use and storage of sensitive data, delineate tasks appropriate for AI technologies against those that must be performed by humans, and determine whether the use of AI technology should be mandatory or integrated in treatment guidelines. However, regulation should not limit therapists’ treatment flexibility and hinder implementation. Future research is needed to better understand how regulatory frameworks can support the integration of AI while preserving ethical standards and the clinician’s role in therapy. Balancing these concerns is therefore key for fostering both ethical safeguards and innovation. Furthermore, the literature on AI in healthcare is largely dominated by data protection issues and ethical concerns^7,29^. In contrast, our discussions with non-AI experts in this field, namely therapists and patients, suggest that these topics do not fully align with their perceived issues. This mismatch may indicate either a need to improve AI literacy so that academic concerns become more salient in practice, or the need for a more fundamental understanding whether current academic discussions, e.g., on ethical issues around algorithmic bias, are insufficiently aligned with the practical needs and lived experiences of end users. It also shows that although some subcategories were referenced less frequently, they may hold important implications, as they could point to potentially relevant future developments. A further explanation for the discrepancy between literature and non-experts may lie in the use of the NASSS framework itself: as an implementation-focused framework, it does not explicitly prompt for more abstract or philosophical ethical considerations, which may have contributed to such issues remaining experientially latent rather than empirically salient in our material.

To move from insight to action, our findings point towards a stepwise roadmap for the practical implementation of AI in psychotherapy. In the initial phase, piloting AI tools for mild conditions can allow both patients and therapists to gain experience, evaluate usability, and ensure alignment with treatment goals. The next phase could then involve designing co-use protocols, integrating AI as a supplementary tool alongside human therapists, thereby maintaining the crucial human element in therapy while leveraging technology for support and continuity. Finally, broader scaling-up efforts should be guided by organizational strategies and policy incentives, such as clear reimbursement schemes, staff training, ethical questions, and infrastructure support, to ensure sustainable and equitable adoption across diverse settings.

This study has several limitations: First, the sample was imbalanced, with three focus groups with therapists and only two with patients. This occurred because participant recruitment followed the principle of data saturation^74^, meaning that data collection continued until no new topics emerged. Additionally, we controlled for theoretical saturation which was achieved once relevant constructs had been identified for all domains of the NASSS framework^74^. The therapist focus groups were conducted first due to earlier sign-ups resulting in fewer patient focus groups, as these started later in the process, and both theoretical and data saturation was achieved after five focus groups. However, given this slight imbalance, future research should include a more balanced amount of groups or conduct additional studies with patients. In interpreting the findings, it is also important to consider how the composition of the focus groups may have influenced the analytic emphasis. As therapist perspectives constitute a larger proportion, issues related to their clinical practice, organizational routines, and system-level considerations may be more prominently represented than aspects grounded in patients’ lived experiences. As data collection with therapists occurred earlier and the analysis proceeded chronologically, the points raised by therapists may have unconsciously shaped an analytic orientation within which subsequent patient data were interpreted. In addition, the analytic orientation provided by the NASSS framework revolves primarily around professional and organizational contexts. This may have further shaped how certain themes were foregrounded during analysis.

Second, our relatively young study sample may reflect a more technology-engaged group^33,62^, potentially skewing perceptions and indicating a sampling bias. Future research should thus target older age groups and patients from lower socioeconomic backgrounds to get a more nuanced understanding of the needs of these often-underrepresented groups. Third, our study only included participants from across Germany; hence, their experiences and perceptions regarding the digital infrastructure and healthcare systems may not be generalizable to other countries. Healthcare policies and technological advancements differ widely across regions, with countries like the U.S. and China leading in AI development for mental healthcare^8^. These regional variances in healthcare systems and regulations may influence how AI technologies are perceived and adopted into mental health treatment, limiting the generalizability of our findings to other contexts with different healthcare frameworks. Fourth, the broader study framing of AI-enabled treatment tools encompasses a heterogeneous range of technologies. Although this limited the specificity of the findings, it allowed us to explore barriers and facilitators across different technologies.

Overall, the study highlights that AI adoption in mental healthcare is a complex and multifaceted issue, influenced by the subjectivity and heterogeneity of mental illnesses, and the diverse therapeutic approaches and workplace environments. Participants’ needs, viewed as facilitating factors, emerged in every domain of the technology adoption process, and when appropriately leveraged, can ease AI adoption. However, given the reservations about AI in mental healthcare, it is essential to design AI solutions that not only align with user needs but also alleviate their concerns. Addressing these barriers proactively holds the potential to overcome hesitation, realize the full potential of AI technology, and consequently, improve mental healthcare by mitigating the treatment gap.

## Supplementary information

Supplementary Information

## Data Availability

The discussion guide, COREQ checklist and the description of AI and AI-enabled technologies in psychotherapy given to participants prior to the study is available on OSF (https://osf.io/9rt52). The codebook and exemplary codes supplementing the study results are available in the online Supplementary Information.
