# Supplementary material for: Navigating the complexity of AI adoption in psychotherapy by identifying key facilitators and barriers
Source: Npj Ment Health Res. 2026 Mar 7;5:17. doi: 10.1038/s44184-026-00199-1 (PMC12967751; doi:10.1038/s44184-026-00199-1)
Supplement: Supplementary file 1 — Supplementary Information [file 44184_2026_199_MOESM1_ESM.pdf]

Supplementary Information for
„Navigating the complexity of AI adoption in psychotherapy by identifying key facilitators and barriers"

Supplement 1: Codebook

Domain	Category	Definition	Sample quote
**1. Condition**		The nature and complexity of mental health conditions targeted by AI technologies.	
	Type of mental health condition	The specific mental health conditions that AI may target.	*"For depression, I imagine it to be a bit more difficult again […] but for things like anxiety disorders, nervousness disorders, I actually imagine it to be quite helpful."*
	Severity of mental health condition	The range of illness severity that AI may address.	*"I do think that a differentiation should be made and that it is particularly in this borderline area - in this gradation of 'mild, moderate, severe mental illness' - that particular attention should be paid when using such technologies."*
**2. Technology**		The characteristics of AI technologies.	
	Useful technology elements	The specific elements that are considered useful for AI.	*"For example, asking about moods at the most unlikely times. So not just in the morning, how do I feel now, how do I feel at lunchtime and how do I feel in the evening, but also to ask something in between, especially when the routine somehow breaks."*
	Customization to users' needs	The extent to which AI can be tailored to specific users' needs.	*"But yes, if it can't respond to the individuality of the person, I find it very difficult."*
	Supplementary use	The use of AI as an adjunct rather than a replacement for traditional therapy.	*"And I think it's really important […] that it's something supplementary and not something that replaces [other treatments]."*
	Usability for multiple user groups	The extent to which AI is suited for use across different groups.	*"And I also think that it's mainly intended for the younger generation somehow, and as has already been said here, the older ones then end up being forgotten."*
	User-friendliness	The ease and intuitiveness with which users can use AI.	*"I think it should be very intuitive and not so complex, because especially if you have depression or something like that, you just don't have the strength to get into it."*
	Evidence-based effectiveness	Evidence supporting AI's effectiveness in improving mental health outcomes.	*"There should be studies that prove that AI-supported interventions have an effect, which is why it makes sense to use it, but the effect is not as great as psychotherapy from a real person."*

**3. Value proposition**		The value of AI technologies for users.	
	Increasing availability of access	The ability for users to access AI at their convenience.	*“So perhaps the advantage of AI-supported technologies is that they can be used flexibly in terms of location. This could be their advantage for use in practice.”*
	Improved efficiency	Potential improvements in efficiency offered by AI.	*“But especially once a pilot has been completed, you have a greater benefit because you know that there is somehow a system that works well, whether it's that the psycho-education is taken off my hands or certain things like a mood diary, activity diary, can be better integrated.”*
	Shifts in depths of therapy elements	The risk of AI oversimplifying therapy and reducing nuanced insights.	*“But sometimes, um, something only comes out in conversation with the therapist, reveiling that there might be an issue there.”*
	AI as third element	The role of AI as an additional agent during psychotherapy sessions.	*“There is then a third element that may influence the therapeutic relationship. [....] Then we have triangulation, […], which I think is complex. In principle, it's a bit like two therapists working on the same patient.”*
	(Commercial) conflict of interest	The influence of commercial interests in the adoption of AI.	*“Yes, I mean, above all, that everything that involves technology is always linked to some company, then to some profit-oriented company, as a rule. [...] But then some company has all the data from all these very intimate personal things that the clients might somehow come up with or that might be helpful in therapy.”*
**4. Adopters**		User factors influencing the adoption of AI technologies.	
	Affinity for technology	Users’ comfort level and enthusiasm in using AI.	*“I also think that there are some personal preferences of the practitioners. So how much you want to get involved in something like this and deal with it or just see the benefits of taking on this work, which is additional in any case, especially at the beginning.”*
	Training requirement	The need for training to use AI effectively.	*“So I could imagine that it would be helpful if there were really um if there could also be training courses on this, for example.”*
	Lack of control	The potential loss of control over therapy processes due to AI.	*“I also asked myself where I might lack this control over my therapy at some point, i.e. how I would like to handle it or how I would also have an influence on the patient.”*
	Trust and openness	The degree of trust in and openness for AI among users.	*“So, an important point would definitely be openness towards the AI, because I know enough people, and I have to say I know enough people myself, who wouldn't trust an AI so much to involve it in such intimate moments or intimate occasions.”*

	Technology understanding	The knowledge or technical literacy required for effective use of AI.	*"I would just be afraid that if I recommend this to the patient, for example, but then I don't quite understand myself how this or how this comes to the result."*
	Lack of human contact	The absence of social and emotional connection after the integration of AI.	*"So, I wouldn't feel seen or acknowledged at all because I would just know: 'Okay, it's just an algorithm.' Artificial intelligence or not. What I would miss is the living counterpart, emphasis on living."*
	Changes in the importance of conventional psychotherapy	The potential of AI changing the perceived need for conventional therapy.	*"I can only put it plainly: there's more of a concern that they'll think, 'Oh, great. A cheap, easy, and quickly available alternative—then we can cut costs elsewhere.'"*
	Technology dependency	(Over-) Reliance on AI technologies.	*"It should just not serve as a safety behavior for the patient or replace anything or create a dependency."*
	Persistence of safety-seeking/ avoidance behavior	The potential of AI to reinforce users' avoidance of difficult interactions or situations.	*"But it can perhaps also lead to patients starting to avoid contact or the relationship difficulties that only become apparent in therapy through shared experience, so that they don't arise in the first place."*
**5. Organizations**		The readiness and capacity of an organization for AI technologies.	
	Differences in the type of institution	Underlying differences in the type of therapy-providing institutions influencing the use of AI.	*"So, if I work in a clinic or in an outpatient clinic that I don't manage myself, so to speak, then I'm dependent on the decisions of the person in charge. Whereas if I have my own outpatient practice, then I'm more independent and can introduce it more easily."*
	Differences in therapy methods	The compatibility of different therapeutic approaches with the potential use of AI.	*"I believe that behavioral therapy has far fewer problems than psychodynamic psychology in the whole complex that is being discussed here. And I think psychoanalysis is probably completely excluded."*
	Digitalization	The extent of digital infrastructure present in institutions.	*"And I believe that we really haven't yet reached the point in the development of digitalization where it really saves time, where it becomes automatic and where people have internalized it in such a way that they can say, okay, it makes my work easier."*
	Pre-use testing opportunities	The chance to try the AI at least once before use.	*"And I think it would be really important to first create opportunities to try things out, so that you could first test it somewhere on a purely platonic level, try it out yourself, before it is then implemented on a patient, so to speak."*
	Other incentives	Existing services or support structures necessary for adopting AI.	*"So, I also think there should probably be some kind of incentive in advance as to why this should be integrated into the processes."*

	Resource and workload considerations	The resources required for integrating AI.	*"For me, I think this point, this familiarization period, would be very, very important, because I think it's very important for us, but above all for the patients, that you don't somehow spend a whole hour of therapy on it, so to speak, to deal with it first."*
**6. Wider system**		The structure, dynamics, and capacity of the system in which AI technologies are introduced.	
	Cost coverage	The extent to which the use of AI is covered by insurance or other funding sources.	*"There is also the question of whether this can be billed somehow."*
	Economic feasibility	Economic considerations regarding the adoption of AI.	*"I believe that to a certain extent there is also a financial aspect, that on the one hand the money has to be there, and on the other hand the willingness on the commercial side to accept the costs for something like this."*
	Regulations and regulatory environment	Legal and policy frameworks guiding the use of AI.	*"By and large, politics is already behind and is monitoring the whole thing at both national and EU level, for example. And there will have to be restrictions or controls in certain areas of application."*
	Data protection measures	Measures ensuring data protection for the use of AI.	*"Above all, there's the data privacy issue: Who has access to all of this? What happens to the data? How long is it stored? What happens in the event of a hacker attack, or something like that? These are all things that need to be clarified thoroughly in advance. Who holds the responsibility? To what extent is the patient responsible for protecting their own data if their device — the one they use for the app or whatever it may be — gets hacked or stolen? So, for me, that's a major area of concern."*
	Liability	Determining accountability for AI outcomes.	*"Let's say I have suicide prevention software, and it somehow works better, ten times better than a therapist […] [in detecting risks]. But still, sometimes people die by suicide. The question then is: Who is somehow liable? Who bears the risk?"*
	Transformations of existing structures	Shifts in the structures of the mental healthcare system.	*"But we could perhaps become even faster. We could become even more productive. Perhaps we could see even more patients. Treat even more. [...] Whether that is necessarily a good thing remains to be seen."*
**7. Embedding and adaptation over time**		The adjustments in the adoption of AI technologies over time.	
	Speed of implementation process	The time it takes to integrate and adopt AI.	*"So, I think individualized things will take a long time, but for these generalized things, I think it will go very quickly."*

	Ongoing scientific validation	The necessity of ongoing research to validate the efficacy of AI prior to its use.	*“But I believe that this will be used across the board in clinics. This simply requires more research and more data, where it can really be proven on a larger data basis in larger samples. 'Okay. There are effects, that makes sense' and I believe that this is what will certainly be used across the board or more widely.“*
**8. Time of use**		The stage in the treatment process at which AI can be considered useful.	
	Use in diagnostics and first screening	AI technologie's role in the diagnostic and screening process.	*“As a kind of diagnostic assessment, perhaps to replace or somehow support initial sessions or counseling sessions.”*
	Use (during waiting times) before therapy	Utilizing AI technologies to engage patients before formal therapy begins.	*"I was just thinking about the extent to which this could also be used as a kind of bridge. Like, during the long waiting periods before starting psychotherapy."*
	Use between therapy sessions	Utilizing AI technologies for support between traditional therapy sessions.	“If you can now fill the time between therapies, for example in the outpatient sector, then if there is simply time in between, you can fill it somehow, especially with exercises.”
	Use in aftercare	AI technology’s role in providing continued support after therapy completion.	“And also at the end of the therapy simply to support you, so that you now have a small safety net that you can rely on and that you won't be discharged and then you're left standing there.”

Supplement 2: Additional supplementary sample quotes

NASSS Domain	Category	Sample Quotes
**Condition**		
	Type of mental health condition	“If there's […] a button. 'I'm having a panic attack. Help me', and it tells me to do different exercises or whatever, I would take it.” (PA01).<br><br>“I was just thinking about patients with eating disorders who are more of the active type, who sometimes manage to eat somehow, but then walk everything they can late at night. […] Recording that again and displaying it visually, I think, could sometimes really help, because many people don't have it on their screen that they get off a bus stop earlier, for example, in order to walk again. And then they add up the kilometers and everyone is wondering 'How is it possible that the person still hasn't put on weight? It might actually help to track this activity using a device like this and make something of it.” (TH11).<br><br>“I am a bit worried that it could possibly become a new obsession [for patients with eating disorders], that a lot of things would have to be tracked.” (TH14).

	Severity of mental health condition	“I do think that a distinction should be made, especially in this threshold area – in this gradation of ‘mild, moderate, severe mental illness’ […].” (TH115).<br><br>“Research could perhaps first approach the mild, softer areas and see how things are going and how we can do it. And somehow gradually increase this in the direction of more illnesses.” (TH03)
**Technology**		
	Useful technology elements	“For example, asking about moods at the most unlikely times. So not just in the morning, how do I feel now, how do I feel at lunchtime and how do I feel in the evening, but also to ask something in between, especially when the routine somehow breaks.” (TH11)
	Customization to users’ needs	“If it can't respond to the individuality of the person, I find it very difficult.” (PA12).<br><br>“Every panic attack manifests itself differently and what helps one person may not help another at all. You tell one person to concentrate on your breathing and then they start to hyperventilate. You tell the other person to concentrate on your breathing and they calm down […].” (PA03).
	Supplementary use	“I don’t think that AI can currently replace real therapy, regardless of the direction, but I think it can definitely have a supportive effect.” (PA05)
	Usability for multiple user groups	“I also see it that way, so to speak, it is important to have a different perspective for children and young people than for adults or geriatric patients. I can possibly see difficulties there […], due to

		the probably rather limited use of digital media. In other words, I ask myself, are these older patients then excluded? That should of course be avoided." (TH18)<br><br>"I can imagine […] [having AI tools available] for a student […], like the upper middle class, […] because there's simply money behind it and perhaps enough people who are interested and who also understand the benefits." (TH11)<br><br>"[…] I believe that there will also be a large section of the population that you do not deal with because it will then be very complicated to translate into simple language applications or actually offer something in their native language." (TH11)
	User-friendliness	"I think it should be very intuitive and not so complex, because especially if you have depression or something like that, you just don't have the strength to get into it." (PA08)
	Evidence-based effectiveness	"There should be studies that prove that AI-supported interventions have an effect, which is why it makes sense to use it, but the effect is not as great as psychotherapy from a real person." (TH14)
**Value proposition**		
	Increasing availability of access	"So perhaps the advantage of AI-supported technologies is that they can be used flexibly in terms of location. This could be their advantage for use in practice." (PT120)<br><br>"If I imagine: 'Okay, I can fall back on previous therapy content, I'm in a crisis at the moment and can simply call it up', I don't necessarily need the therapist for that, that in turn can also have positive effects in terms of autonomy." (TH18).

	Improved efficiency	"[…] Once a pilot has been completed, you have a greater benefit because you know that there is somehow a system that works well, whether it's that the psychoeducation is taken off my hands or certain things like a mood diary, activity diary, can be better integrated." (TH14).
	Shifts in depths of therapy elements	"Psychologists are used to trying to capture behavior and feelings in numbers in some way. But I wonder if that's too simplistic, because we are actually much more complex than that, more than could be broken down into an algorithm that works when certain words are written, like in chatbots or similar things. I mean, the risk is that we overlook something or miss something, but I think that risk is very high. Especially when it comes to initial contact with psychotherapeutic services, where establishing a relationship is actually really important, and I wonder if that's being functionalized away." (TH02)
	AI as third element	"There is then a third element that may influence the therapeutic relationship. [....] Then we have triangulation, so to speak, which I think is complex. In principle, it's a bit like two therapists working on the same patient." (TH01)
	(Commercial) conflict of interest	"[…] I mean, everything that involves technology is always linked to some company, then to some profit-oriented company, as a rule. [...] But then some company has all the data from all these very intimate personal things that the clients might somehow come up with or that might be helpful in therapy." (TH08)
**Adopters**		
	Affinity for technology	"I also think that there are some personal preferences of the practitioners. So how much you want to get involved in something like this and deal with it or just see the benefits of taking on this work, which is additional, in any case, especially at the beginning." (TH09)
	Training requirement	"I could imagine that it would be helpful if there […] could also be training courses on this […]." (TH04)

		"There also has to be good training for the practices, because when I look at my own practice, which is a small practice in the countryside with, I think, three employees, you have to be able to instruct them well and train them well. And I think it has to be relatively quick, because the practice is pretty full. And there has to be a good way for them [therapists] to learn." (PA01)
	Lack of control	"I also asked myself where I might lack this control over my therapy at some point, i.e. how I would like to handle it or how I would also have an influence on the patient." (TH03)
	Trust and openness	"An important point would definitely be openness towards the AI, because I know enough people, and I have to say I know enough people myself, who wouldn't trust an AI so much to involve it in such intimate moments or intimate occasions." (PA01)
	Technology understanding	"I would just be afraid that if I recommend this to the patient, for example, but then I don't quite understand myself how this or how this comes to the result." (PA06)<br><br>"I could also imagine that some patients simply have very specific questions when this and that appears in the app, what do I have to do? I think it's important to have an answer ready somehow." (TH14)<br><br>"[…] Therapists […] [should] also [be] familiar with it. So that they can also give me recommendations or perhaps explain to me how to handle it or something like that. Um, and that they're not also up to date: 'well, there's an app, you can use it, it's good, but that's all I know about it'." (PA06)

	Lack of human contact	“So I wouldn't feel seen or acknowledged at all because I would just know: ‘Okay, it's just an algorithm.’ Artificial intelligence or not. What I would miss is the living counterpart, emphasis on living.” (PA03)<br><br>“Otherwise I would agree with my colleague that, as enthusiastic as I am about this in other areas, I also fear - or rather hope, as a therapist - that the human aspect is something fundamental, and that people can only truly heal within relationships.” (TH02)
	Changes in the importance of conventional psychotherapy	"I can only put it plainly: there’s more of a concern that they’ll think, ‘Oh, great. A cheap, easy, and quickly available alternative—then we can cut costs elsewhere.’" (PA03)
	Technology dependency	"What I mentioned earlier as a pro argument could also be seen as a con argument here: the shift from independence from the therapist to dependence on a device." (TH18)
	Persistence of safety-seeking/ avoidance behavior	“But it can perhaps also lead to patients starting to avoid contact or the relationship difficulties that only become apparent in therapy through shared experience, so that they don't arise in the first place.” (TH02)
**Organizations**		
	Differences in the type of institution	“At least in my clinic I can imagine that the clinic management or the head physician could be interested in this, because they tend to have such a market-oriented awareness and say that innovations and things like that are good and can certainly be incorporated.” (TH11)<br><br>“So if I work in a clinic or in an outpatient clinic that I don't manage myself, so to speak, then I'm dependent on the decisions of the person in charge. Whereas if I have my own outpatient practice, then I'm more independent and can introduce it more easily.” (TH18)

	Differences in therapy methods	"I believe that behavioral therapy has far fewer problems than depth psychology in the whole complex that is being discussed here. And I think psychoanalysis is probably completely out of the question." (TH06)
	Digitalization	"And I believe that we really haven't yet reached the point in the development of digitalization where it really saves time, where it becomes automatic and where people have internalized it in such a way that they can say, okay, it makes my work easier." (TH09)
	Pre-use testing opportunities	"And I think it would be really important to first create opportunities to try things out, so that you could first test it somewhere on a purely platonic level, try it out yourself, before it is then implemented on a patient, so to speak." (TH09)
	Other incentives	"I also think there should probably be some kind of incentive in advance as to why this should be integrated into the processes." (TH04)<br><br>"I would need someone to help me […], a point of contact, some kind of hotline or whatever, where I could ask if any problems arise." (PA11)
	Resource and workload considerations	"For me, I think this point, this familiarization period, would be very, very important, because I think it's very important for us, but above all for the patients, that you don't somehow spend a whole hour of therapy on it, so to speak, to deal with it first." (TH17)
**Wider system**		
	Cost coverage	"There is also the question of whether this can be billed somehow." (TH02)

		“It would also be important for me that if this […] [technology] costs anything […] that it would be financed and covered by the health insurance company in any case and in a reasonably uncomplicated way” (PA06).
	Economic feasibility	“I believe that to a certain extent there is also a financial aspect, that on the one hand the money has to be there, and on the other, there also needs to be a willingness, especially from the business side, to accept the costs for something like this” (TH14).
	Regulations and regulatory environment	“By and large, politics is already behind and is monitoring the whole thing at both national and EU level, for example. And there will have to be restrictions or controls in certain areas of application.” (TH06)<br><br>“If this is always linked to any guidelines, then it may be that the whole thing becomes quite a cage as far as the application is concerned.” (TH09)
	Data protection measures	“Above all, there's the data privacy issue: Who has access to all of this? What happens to the data? How long is it stored? What happens in the event of a hacker attack, or something like that? These are all things that need to be clarified thoroughly in advance. Who holds the responsibility? To what extent is the patient responsible for protecting their own data if their device — the one they use for the app or whatever it may be — gets hacked or stolen? So, for me, that’s a major area of concern." (PA03)<br><br>"At the same time, there's also the issue of data privacy again, as you mentioned earlier: How is something trained, using which data, and how do I protect my clients when they find themselves in certain situations? What goes into the health record? What gets documented, and where? How is the data processed? Who has access to it? And what does the healthcare system do with

		it? There’s definitely the potential for risks — for example, increased transparency that could lead to mental health being treated as a different kind of commodity." (TH15)
	Liability	“Let's say I have suicide prevention software and it somehow works better, ten times better than a therapist could recognize. But still, sometimes people kill themselves. Then the question is, who is somehow liable? Who bears the risk?” (TH08)
	Transformations of existing structures	“But we could perhaps become even faster. We could become even more productive. Perhaps we could see even more patients. Treat even more. [...] Whether that is necessarily a good thing remains to be seen.” (TH10)
**Embedding and adaptation over time**		
	Speed of implementation process	“Individualized things will take a long time, but for these generalized things, I think it will go very quickly.” (PA12)<br><br>“When I look at the discussions currently happening in Germany regarding the new Psychotherapy Law and Training Act, where it’s still not really clear how and by whom it will be funded, I think to myself: ‘This will probably take decades until Germany might actually reach the point where they say: ‘OK, AI-supported psychotherapy, […] maybe. But only if all the framework conditions are 100% perfectly balanced and clarified. Personally, I have to admit, it’s hard for me to judge. However, I believe […] that, unfortunately, these topics likely do not have the highest priority on various political agendas. And that’s why it will just keep getting delayed even further." (PA05)<br><br>“And I don't think it's possible to estimate just how, how rapidly the development will ultimately take place” (PA05).

	Ongoing scientific validation	“This simply requires more research and more data, where it can really be proven on a larger data basis in larger samples. 'Okay. There are effects, that makes sense' and I believe that this is what will be used across the board or more widely across the board.” (TH14)
**Time of use**		
	Use in diagnostics and first screening	“As kind of diagnostic assessment, perhaps to replace or somehow support initial sessions or counselling sessions.” (PA02)<br><br>“And would diagnostics be feasible for you? For me, that's already where it ends. Sure, it can do these tests or questionnaires or whatever. But what I actually want to say is that even diagnostics can't be done by AI." (TH16)
	Use (during waiting times) before therapy	“Especially with the waiting times. That this [AI technology] may have a positive effect on patients being given the chance to work on the issues directly or at an earlier stage, or to get in touch or have a consultation or conversation.” (TH03)<br><br>“Or you've already been to umpteen initial consultations and haven't yet found a fixed place where you can simply get what you've been prescribed by your doctor or by therapists, psychiatrists etc.” (PA12)<br><br>“But I think, especially in the initial phase, it's really not helpful to talk to something that's not personal. So I think it's very, very important to have personal contact [to a human therapist] in the initial phase.” (PA02)
	Use between therapy sessions	“If you can fill the time between therapies, for example in the outpatient sector. Then if there is simply time in between, you can fill it somehow, especially with exercises.” (TH13)

	Use in aftercare	“And also at the end of the therapy simply to support you, so that you now have a small safety net that you can rely on and that you won't be discharged and then you're left standing there.” (PA12)
